# Patients’ experiences of motivation, change, and challenges in group treatment for insomnia in primary care: a focus group study

**DOI:** 10.1186/s12875-018-0798-2

**Published:** 2018-07-09

**Authors:** Christina Sandlund, Kimberly Kane, Mirjam Ekstedt, Jeanette Westman

**Affiliations:** 1 Department of Neurobiology, Care Sciences and Society, Karolinska Institutet, Stockholm, Sweden; 2 Academic Primary Health Care Centre, Stockholm County Council, Solnavägen 1 E, Box 45436, 104 31 Stockholm, Sweden; 3 Aging Research Center, Karolinska Institutet and Stockholm University, Stockholm, Sweden; 4 Department of Health and Caring Sciences, Linnaeus University, Stagneliusgatan 14, SE-392 34 Kalmar, Sweden; 5 Department of Learning, Informatics, Management and Ethics, Karolinska Institutet, Stockholm, Sweden

**Keywords:** Behavioral change, General practice, Group therapy, Health behavior, Motivation, Nursing, Patient education, Self-efficacy, Sleep initiation and maintenance disorders

## Abstract

**Background:**

The majority of patients who seek help for insomnia do so in primary health care. Nurse-led group treatment in primary care based on cognitive behavioral therapy for insomnia (CBT-I) can lead to improvements in both day- and nighttime symptoms. This study aimed to explore patients’ experiences of nurse-led group treatment for insomnia in primary health care.

**Methods:**

Seventeen patients who had participated in the group treatment program were interviewed in five focus groups. Interview transcriptions were analyzed with qualitative content analysis.

**Results:**

Four themes emerged that described patients’ experiences of the group treatment program. *Involvement and trust open the door for change*: Motivation to engage in treatment arose from patients’ own desire for change, from being together with others who shared or understood their struggles, and from feeling emotionally affirmed and trustful. *Competence arising from deeper understanding*: Patients obtained knowledge and made it their own, which enabled them to develop functional sleep habits and let go of sleep performance and worry. The ability to impact their insomnia increased patients’ trust in their own efficacy and helped them persist in behavioral change. *Struggling with vulnerability and failure*: Treatment was tough, and patients could feel challenged by external circumstances. Moreover, they could distrust their own efficacy. *Tailoring treatment to individual needs*: Patients experienced different life circumstances and adapted the techniques to their needs and abilities by focusing on what felt right for them.

**Conclusions:**

Patients went through a process of motivation, change, and challenges. They experienced certain aspects of treatment as essential to changing behavior and achieving improvements. Examples included being in a group with others who shared similar experiences, gaining knowledge about sleep, keeping a sleep diary, and practicing the sleep restriction technique. The study provides insights into patients’ struggles during treatment, both those related to external circumstances and those related to feelings of vulnerability and failure. It also highlights the importance of adapting treatment to patients’ differing needs, underscoring the value of person-centered care.

## Background

Sleep plays a vital role in good health and well-being and living with insomnia affects overall health. Its impact on health is reflected in physical and mental illnesses, cognitive decline, and reduced quality of life [1–3]. Nine to 15% of people worldwide have insomnia [4, 5], which is experiential in nature and diagnosed on the basis of a person’s own perceptions of sleeping difficulties and the daytime impairments they associate with these difficulties [6, 7]. Qualitative studies show that people who live with insomnia can experience impaired cognitive, emotional, and physical functioning on a daily basis, as well as limitations in work performance, social participation, and life aspirations [8]. It is these experiences that cause people to seek treatment, typically in primary care [9]. Patients who seek primary health care for insomnia commonly receive hypnotic drugs even though the recommended treatment is cognitive behavioral therapy for insomnia (CBT-I) [10, 11].

Because of the experiential nature of insomnia, qualitative evaluations can provide insight into treatments that quantitative evaluations cannot, illuminating patients’ experiences of the treatment, symptom change or stability, and processes through which improvements did or did not occur. Such explanations for how and why psychological and behavioral treatments work are important, as a better understanding of key mechanisms can facilitate the design of more effective and efficient therapies [12, 13].

Few studies have explored patients’ experiences of insomnia treatment. Most qualitative studies on insomnia focus on people’s experiences of living with the disorder, professionals’ experiences of treating patients with insomnia, and patients’ experiences of health care professionals’ attitudes toward the disorder and its treatment [14]. However, one study has explored patients’ experiences of long-term treatment with hypnotic drugs [15]; another, patients’ experiences of mindfulness-based group treatment for insomnia [16]; and a third, patients’ experiences of sleep restriction therapy, one technique included in CBT-I [17]. On the other hand, there have been numerous explorations of patients’ experiences of individual psychotherapy for problems other than insomnia [18]. Some have focused on experiences of treatment-related change [19, 20] and others on helpful [21, 22] or unhelpful aspects of treatment [23, 24].

Although CBT-I is the recommended treatment for insomnia, primary care patients’ access to it is limited. We therefore conducted a randomized control trial in Swedish primary health care to investigate whether a nurse-led, CBT-I based group treatment program for insomnia could be effective in treating patients with insomnia who seek primary care [25]. The 2011-2104 trial included 165 patients with insomnia aged 20 to 90 years (mean age 54 years), mostly women (72.7%). Analyses showed that the group treatment significantly improved insomnia symptoms (insomnia severity, sleep, and daytime symptoms), and decreased hypnotic drug use [25, 26]. However, some patients improved more than others, and some may not have improved at all. Because qualitative approaches can illuminate the lived experiences behind quantitative results [27], we reasoned that a qualitative study could potentially reveal patients’ experiences of key mechanisms that underlay the quantitative findings, helping us improve the efficiency of the intervention.

In the present study, we thus aimed to explore patients’ experiences of the nurse-led group treatment program for insomnia in primary health care.

## Methods

### Study design

We performed focus group interviews with patients who had participated in the nurse-led group treatment program for insomnia in primary health care. The interviews took place in Stockholm County, Sweden, from 2013 to 2015. Focus groups promote the expression of differing views, which can lead to further discussion and exploration of experiences [28]. Qualitative content analysis as described by Graneheim and Lundman was used to analyze the interviews [29, 30]. This method is appropriate for illuminating lived experience and has a well-defined analytical process and clear concepts for discussing trustworthiness [30].

### Participants

Seventeen patients who had taken part in the group treatment program participated in this study. To participate in group treatment, patients had to be 18 years or older and meet the criteria for insomnia disorder [31]. Inclusion and exclusion criteria are described in detail elsewhere [25]. Patients who participated in the focus group interviews had been in the control group in the randomized controlled trial [25] and received treatment as usual (mainly hypnotic drugs) while waiting three to 4 months for group treatment.

All focus-group participants had attended treatment together and thus knew one another. We expected patients’ shared experiences of treatment and familiarity with each other to facilitate group discussions [28]. All patients who participated in treatment between 2013 and 2015 were asked to take part in the focus groups (*n* = 23).

Five focus groups were conducted. Each group included three or four patients, and all were women. Their median age was 57.9 years (25 to 75 years). Nine were employed and eight were retired. Their mean Insomnia Severity Index score before treatment was 18.9 (range 14-25; possible scores 0-28) [32]. Scores above 11 indicate clinical insomnia [33].

Five patients (four women and one man) who had participated in the group treatment did not take part in the interviews. Three were sick on the day of the interview, one was traveling, and one was taking care of a sick relative.

### Group treatment program for insomnia

Patients met at their primary health care center for seven 2-h group treatment sessions over the course of 10 weeks. The first six sessions were weekly and were followed by a final session 4 weeks later. The group treatment program was based on the theoretical framework and techniques of CBT-I [32, 34–36]. CBT-I targets mental and physical hyperarousal, maladaptive cognitions (e.g., rumination), and maladaptive behaviors (e.g., irregular sleep habits) [37]. It includes educational components and behavioral and cognitive techniques that aim to change these cognitions and behaviors, improve sleep, and increase ability to cope with insomnia [38].

Educational components of the program included information about sleep and sleep regulation and theoretical frameworks for understanding insomnia. Patients kept a sleep diary throughout the program and used their entries as the basis of reflections during group sessions. At each session, the nurse who led the group introduced one or more techniques that patients applied as homework between the sessions (relaxation, worry time, paradoxical intention, sleep restriction, stimulus control, reduction of hypnotic drugs, sleep hygiene, identifying and arguing against unhelpful thoughts, stress reducing techniques, and techniques to cope with daytime symptoms). At the last session, each patient identified treatment gains and created an individual program to prevent relapse and maintain benefits. A comprehensive description of the treatment program is found elsewhere [25].

The nurses who led the groups were district nurses, registered nurses with a 1-year master’s degree in primary health care nursing. Together with physicians, district nurses make up the largest group of professionals in Swedish primary care. In partnership with patients and in close collaboration with other health care professionals, they work to meet patients’ basic needs (e.g., sleep and rest), promoting health, preventing illness, and alleviating suffering [39]. The nurses who led the treatment program had taken part in a 16-h training course that covered the causes and diagnosis of insomnia and how to deliver the treatment. None had previous experience of CBT-I, but all were experienced in nursing (median 29 years).

### Focus group interviews

Each interview took place at the end of the last treatment session (session seven). In two of the focus groups (groups 2 and 5), the moderator was also the nurse who had led the treatment sessions (CS). In the other groups, the nurse who led the group left prior to the start of the interview. Each interview took approximately 70 min and was audio recorded and transcribed verbatim.

The moderator (CS) used a semi-structured interview guide with open-ended questions. The guide was developed for the study. The interview started with opening questions, such as “Can you tell me about how it was before you started the group treatment?” and “Can you tell me about a night, how it was, how it felt, how it affected you during the day, and what you did so you could sleep?” The moderator then used the following questions to guide the interviews: 1) “How do you experience your sleep after the treatment?” 2) “Is there anything special that helped you?” 3) “What changes have you made?” 4) “What was it that got you to make these changes?” 5) “Was there something that didn’t work so well?” 6) “Was there something about the treatment that you thought was hard? That didn’t fit you? Why do you think that was?” and 7) “What do you think could be done to improve this program?” Probing questions and statements were used to clarify or deepen the discussion, including, “What did you do then?” “What could have helped you to act or feel otherwise?” “What do you think that was due to?” and “Tell me more about that.”

### Data analysis

The transcriptions were analyzed using qualitative content analysis [30]. Patients’ experiences were explored at a manifest and descriptive level close to the text and a latent, more abstract and interpretative level [29, 30].

We took an inductive approach [40], generating our ideas on the basis of the interview texts. In the beginning of the process, we read the texts several times to get a sense of the whole. After reading, we divided the text into meaning units that were relevant to the aim. Next, we shortened the meaning units to condensed meaning units, taking care to preserve the core meaning and context. These condensed meaning units were labeled with codes that expressed the content of the meaning unit. The codes were then sorted on the basis of similarities and differences and abstracted into conceptual categories. Twelve subthemes emerged from the categories and were abstracted to four themes that captured the essence of patients’ experiences of the group treatment for insomnia. Throughout the entire analytical process, we moved back and forth among all levels of analysis, from the original text to the themes.

The researchers took several steps to achieve trustworthiness. CS and KK each conducted their own initial coding and analysis of the transcribed interviews before coming together to compare their findings. All researchers engaged in ongoing discussion and reflection throughout the process of analysis until consensus was reached. Prior understanding of insomnia, nursing, and the intervention were important topics of the ongoing discussions throughout the entire analytical process.

## Results

Four themes emerged that described patients’ experiences of the group treatment: involvement and trust open the door for change, competence arising from deeper understanding, struggling with vulnerability and failure, and tailoring treatment to individual needs. The themes and the subthemes that lay behind them are presented in Table 1.

**Table 1:** Overview of subthemes and themes

Subthemes	Themes
Desiring positive change	Involvement and trust open the door for change
Being together with others pushes you forward	
Feeling emotionally affirmed and trustful	
Obtaining knowledge and making it your own	Competence arising from deeper understanding
Developing functional sleep habits	
Letting go of sleep performance and worry	
Trusting in own efficacy	
Finding treatment tough	Struggling with vulnerability and failure
Challenged by external circumstances	
Distrusting own efficacy	
Experiencing different life circumstances	Tailoring treatment to individual needs
Focusing on what feels right	

### Involvement and trust open the door for change

#### Desiring positive change

Patients experienced a desire for positive change grounded partly in health concerns, such as the belief that high blood pressure could be a result of insomnia. The non-pharmacological nature of the treatment could also lie behind patients’ engagement. They said that it felt appealing that the group treatment addressed their overall problem and that they saw the opportunity to escape the need to take hypnotic drugs for their entire life. Moreover, patients described having come to the end of their rope. They felt they had tried everything, and it had not solved the problem.

I think it was that I gave up, that I'm so tired of it, and that it's gone on so awfully many years, and that I felt that I had tried everything except the strongest sleeping pills. That yeah, now it's the last chance, like: let's do this. (Patient [P] 3, Group [G] 5)

Moreover, patients felt a commitment to themselves. Once they chose to do something about their problem, they felt obligated to follow through.

#### Being together with others pushes you forward

Patients described how meeting in person and having someone who led them was supportive. It pushed them forward, helping them initiate and persist in behavioral change. They also felt a commitment to the group which pushed them toward behavioral change.

And once you've started in this kind of a group, you can't let it down. Instead, you have to do your bit. If you had done it by yourself, you might have thought that no, this was too tough. But if you start it, you have to complete it, so to speak. (P1, G2)

#### Feeling emotionally affirmed and trustful

Patients found that living with insomnia in a world of good sleepers could be lonely; meeting others who had the same problem or who understood it made them feel less “weird.” In the group treatment context, where they were surrounded by people who knew what it was like to live with insomnia, the patients felt understood and less lonely: “We, like, experienced each other’s wakeful nights and everyone just, ‘God, I know, I know, it’s terrible, it’s terrible!’ And then when someone had slept more, it was like everyone just, ‘Oh, it’s unbelievable, oh!’” (P3, G3). Patients also experienced engagement, both from other group members and the group leader, and thus felt taken care of and seen. The small size of the groups helped patients feel heard. Feeling involved and feeling that others were involved helped them go on with treatment.

Trust in authorities (society, primary health care, and primary health care professionals) helped motivate patients to take part in the treatment. As the primary health care center was offering this program, they thought it must be good. Moreover, patients found it convenient and motivating to meet at their own health care center because they felt at home there. If their doctor had suggested the treatment, patients wanted to follow their doctor’s advice. Their trust in the group leader let them dare to “go for it” and try new behaviors.

### Competence arising from deeper understanding

#### Obtaining knowledge and making it your own

Patients experienced knowledge as a key ingredient in treatment. They gained this knowledge via the educational components of the treatment and particularly highlighted the importance of learning about sleep and how it works. Patients said they were familiar with some of the information presented but came to understand it at a deeper level during the course of treatment.

The introduction of techniques one at a time kept patients from feeling overwhelmed, and by practicing the techniques, patients learned what worked for them. This kind of learning by doing allowed them to try new paths and break old patterns. Their intellectual learning was strengthened by the bodily experience that came as a result of practicing the techniques, which confirmed what they had learned about the sleep process. One patient expressed it this way: “You got your body to cooperate purely physically, it wasn’t just up here, instead the physical, too, that got you to understand, your body to understand” (P2, G2).

Patients described how repeating and discussing information and practicing techniques over the 10-week treatment period helped them process the information and achieve deeper understanding. By sharing experiences with each other, the patients gained new perspectives: “It’s a little like brain washing. You repeat things, and we sit here, and you tell us, and we talk about it here, and then we repeat things the whole time. And finally, it sinks in somehow” (P1, G3).

Moreover, patients said they gained perspective and achieved insight by reflecting on their own thinking and behavior. For instance, one described the realization that it was her mind rather than her body that caused her sleep problems. The techniques of worry time and identifying and arguing against unhelpful thoughts helped patients in this process. Additionally, keeping a sleep diary helped the patients reach a deeper level of understanding by giving them an overview of their behavior. “It’s the sleep diary, I feel, that makes it sink in. Because you document it. And you see some kind overarching picture of your behavior. Otherwise you just muddle around” (P3, G3).

#### Developing functional sleep habits

Patients described achieving more functional sleep habits during treatment. Practicing techniques (e.g., sleep restriction) enabled them to break old patterns of unhelpful sleep habits, and by trying out what worked for them, they began to adopt new sleep habits in their daily lives. One of these was a more regular sleep schedule. Another was spending less time in bed. They felt that these new habits consolidated their sleep, and that the more efficient sleep meant they were not as tired during the day.

Sleep quality has become totally different. I'm up at over 80% [sleep efficiency] now. I'm really very satisfied about this, and I go to bed, somehow, I've found that I got to bed between 12:00 and 1:00 and usually wake up around 6:00. And it works, and I'm not tired during the day. (P4, G3)

They found that spending less time awake in bed left less room for worry and rumination and talked about how going to bed later felt liberating and gave them more spare time. Patients also cut down on the time they spent sleeping during the afternoon. Instead they took short daytime naps. These naps could have a positive impact on nighttime sleep and could make them feel more alert. Moreover, taking short naps facilitated a more regular nighttime sleep schedule and reduced worry.

Yeah, that power nap is probably pretty good for me. It feels like a little reward there in the middle of the day before 3:00, that, “Now I'm actually a little tired. I do this now, and I'll feel better. When I pick up the grandkids, then I'm a little more alert.” (P3, G2)

#### Letting go of sleep performance and worry

Patients talked about how they became able to dedramatize sleep and insomnia by accepting the situation and letting go of the idea that they had to force themselves to sleep. By gaining perspective on their sleep problems, the pressure they put on themselves to achieve perfect sleep diminished. For instance, one said, “‘Screw it, I don’t have the energy to be angry anymore.’ And then it just let go, there was something that let go then” (P2, G2). Patients also grew more relaxed in their attitudes toward feelings of tiredness and sleepiness. They talked about how before group treatment, tiredness and sleepiness felt entirely negative. Now these feelings were less like enemies and more like allies that could help them sleep well. Moreover, they were able to let go of sleep-related worries because their perspectives on sleep had become more relaxed.

It was a really big relief for me, this insight that it's not dangerous to lie awake, and it’s not dangerous if I only got five hours. It's given me a much more relaxed way to handle it all. So it made a really big difference. Because before I could stress myself: “God, now I'm going to get a migraine. Oh God, oh!” I lay there and my thoughts ran around in circles and got more and more irritated and stressed that I couldn't sleep, and then of course you can't sleep. I often lay awake like that. But I think that that this part has been a huge ah-ha help for me. (P4, G4)

Knowing that they had strategies reduced worry. For instance, one patient said that just knowing she could choose to get up for a while if she could not sleep made her less worried about sleeplessness. Moreover, the relaxation exercise helped patients achieve mental and bodily relaxation, and worry time helped them gain perspective on worry. Patients said they developed routines, and in doing so, experienced how the routines let the body meet its needs, which in turn made it easier to handle worry and relax. Developing routines resulted in a more regular amount of sleep every night, which gave patients a feeling of flow and energy. They could experience relief by focusing on practical matters rather than feelings and relying on routines instead of trying so hard emotionally.

**P3**: But now I have a good flow, I sleep. I have these routines. Then maybe I don't sleep so much, it's just five hours, but even so, it's given me some kind of overall energy that's good. . . . So that's what I feel, that during the actual night I've been able to handle my worry a little more, thanks to the fact that my body got its needs met with routines and all. It's been a nice insight—that like the body needs its harmony.

**P2**: The body really likes its routines. That's become really clear.

**P3**: Exactly. The body likes it, and it also becomes, it's nice that you don't need to influence everything, to strain your brain the whole time.

**P2**: Exactly. That's what I think, too.

**P3**: There's so much of that the whole time. That you can also affect things. I like sleep restriction. It's a good thing. (G5)

#### Trusting in own efficacy

Patients described an increased trust in their own efficacy. They experienced the power to act and positive feelings that were related to doing what they could: “Every time you manage to change something, you feel more like life is in your own hands” (P3, G3). Patients achieved trust in their own ability to sleep, including their body’s ability to take care of its sleep needs. They found that their bodies could actually produce better sleep than drugs could, which increased their trust in their own efficacy. Patients experienced trust in the techniques; they said they felt confident in having strategies that worked. They found it helpful that the sleep restriction technique was introduced early in the treatment. It quickly had a positive effect on sleep, and the patients felt that this put them on the right track. Once they were on the right track, experiencing that “it worked” motivated them to keep going. One put it like this: “It’s obvious. When you notice it’s going this way, you keep going” (P3, G4).

### Struggling with vulnerability and failure

#### Finding treatment tough

Patients described how fear of sleeplessness and frustration about sleeplessness could interfere with the ability to stop trying to force sleep to come, to relax, and to take action and use the techniques they had learned. Physical tiredness and mental exhaustion could make it more difficult to handle problems: “It’s hard to deal with things like this when you’re already tired and exhausted, so then you’re also more sensitive” (P2, G5).

Some difficulties were related to the sleep restriction technique, which limited patients’ hours in bed and initially caused sleep deprivation, making it difficult to adhere to the technique. One patient explained: “I was going to go to bed at 1:00 at night and get up at 6:00. But I tried to keep to that as well as I could, but it hasn’t gone very well for me. I was really tired sometimes, and I’ve gone to bed a little earlier” (P3, G1).

#### Challenged by external circumstances

Patients described how circumstances outside their control could make it difficult to put the techniques into practice. For instance, one patient had problems with pain. Another explained that her partner was unstable on his feet. She had trouble relaxing, as he got up several times a night and she was worried that he might fall. The environment could also make it challenging to use techniques. If it was hectic and noisy at work, for example, it could be difficult use the relaxation exercise.

The strict routines that came with sleep restriction could make it difficult to live a life that included spontaneous social events, such as staying up late with friends and/or sleeping over at someone else’s house. One patient said that the late bedtime made her start eating during the evening, which she experienced as a problem.

#### Distrusting own efficacy

Patients could express feelings of uncertainty. They described how techniques that usually worked sometimes might not, for instance in extra stressful situations. “Like last week, work was like a disaster with tons of conflicts and pressure, and then I can lie down and breathe as much as I want, but it [relaxation] won’t come” (P2, G5). Even though patients found techniques for coping with worry helpful, it might tend to come back. Moreover, they could feel that improvements were fragile and the future uncertain: “It feels fragile. What if it comes back, that it starts to be hard to sleep?” (P1, G5).

Patients could also express feelings of helplessness and failure, for example if they felt they had not improved as much as others or had not reached their own treatment goals. They could find that even though they did everything right, their situation did not improve. For instance, one said that even though she had no specific worries and had adopted functional sleep habits, her sleep did not get better. She attributed this failure to improve to herself: “So I don’t have any reasonable explanation except that there’s something that’s wrong in my brain” (P1, G2).

### Tailoring treatment to individual needs

#### Experiencing different life circumstances

Depending on their life circumstances, patients could view techniques as more or less important. For example, those who worked sometimes wanted more discussion about how to cope with stress, whereas those who were retired might not recognize themselves in the discussion about stress. Moreover, patients could find it stressful to repeatedly leave work early to get to the treatment sessions on time. On the other hand, some could find the multiple short sessions comfortable.

#### Focusing on what feels right

Patients felt they had the freedom to choose from a smorgasbord of techniques that included something for everyone: “But we’ve been helped by so many different things, too. So it’s probably that everyone, yeah, everyone can, like, find something” (P3, G3). They focused on what felt right for them, trying things out, choosing what suited them, and using techniques in the way that worked for them. They adapted and adjusted the techniques to fit their own needs, life situations, abilities, and inclinations. Patients also described how the group leader adapted the techniques to fit the needs and circumstances of each individual. For example, patients felt that adjustments to the sleep restriction schedule made it easier to adhere to sleep restriction.

## Discussion

This qualitative analysis revealed four themes that illuminate patients’ experiences of a nurse-led group treatment for insomnia in primary health care [25]: involvement and trust open the door for change, competence arising from deeper understanding, struggling with vulnerability and failure, and tailoring treatment to individual needs. To the best of our knowledge, this is the first study to investigate patients’ experiences of multi-component CBT-I-based group treatment for insomnia.

### Discussion of findings in relation to previous research

Readiness for change can be an important step toward change [41]. In this study, patients started treatment with a desire for change, and this desire may have influenced their engagement in the treatment and their ability to achieve improvements [42, 43]. Patients described how they felt “strange,” “weird,” and “lonely” in a world of “good sleepers.” Such feelings of isolation and alienation have been described previously by people living with insomnia [8]. In the current study, important motivators for engaging in behavioral change were feeling that you were not alone in your problems and that you had the support of other group members who shared the experience of living with insomnia. These findings echo those of previous studies of patients’ experiences of mindfulness-based group treatment for insomnia [16] and of group treatment for health problems other than insomnia (obesity and diabetes) [44, 45]. Moreover, in our study, patients found that the engagement and understanding of the group leader motivated them to try new behaviors. The importance of a trustful relationship with care providers for behavioral change and health outcomes is well known, both from qualitative [16, 18] and quantitative studies [46, 47].

Our finding that the group format was important to behavioral change is in line with the findings of previous studies [48, 49]. A set of therapeutic or curative factors common to group therapy seem to be the main mechanisms behind such change [50], and many of these factors are recognizable in our findings. They include being in a safe and secure environment in which people can both listen and be heard; feelings of involvement, trust, and validation; recognizing that one’s own experiences are similar to those of others; becoming more optimistic about one’s own chances of success after witnessing the success of others; learning by observing the journeys of other group members; gaining knowledge from the group members and leader; gaining insight through the feedback of others; and gaining insight into what lies behind one’s own feelings and behaviors [50].

Educational theory posits that lived experience is crucial to learning [51] and that learners move from gaining fact-based knowledge through experience and reflection to deeper understanding and the ability to broadly apply what they have learned [52]. Consistent with educational theory, patients in our study experienced knowledge as essential to behavioral change. They described how repeated information and discussion, shared experiences, keeping a sleep diary, and bodily experience that came from practicing techniques facilitated a deeper understanding of sleep and their own behaviors. Previous studies of group treatment [45] and of individual treatment [18] have also found that such deeper understanding facilitates behavioral change. Like our findings and in keeping with educational theory, the findings of a previous study of sleep restriction underscore the role that bodily experience plays in deeper understanding [17]. Similar to the patients in our study, patients in that study found that sleep restriction led to bodily experiences congruent with what they had learned about sleep and noticed the quality of their sleep was better.

In the present study, patients’ deeper understanding of sleep and of their own behaviors made them feel competent. Feelings of competence helped patients relax and let go of sleep performance and worry, develop more regular sleep habits, and reduce the time they spent in bed. These findings are clinically important because worry—especially worry about sleep—perpetuates insomnia by inducing maladaptive sleep behaviors aimed at achieving sleep and avoiding sleeplessness, such as spending extra time in bed [34].

Our findings that feelings of involvement, trust, and competence were important to motivation and to patients’ success in changing their behaviors are consistent with behavioral theory [53, 54]. These feelings are similar to those of autonomy, relatedness, and competence described in self-determination theory as crucial to the intrinsic motivation that allows people to take action and persist in behavioral change [54]. Moreover, according to the theory of self-efficacy, feelings of competence are important to motivation, and self-efficacy is crucial to how people choose to act and how they cope with challenges [53].

In addition to feeling involved, trustful, and competent, patients could also struggle with feelings of vulnerability and failure. Some vulnerabilities may have been related to insomnia. When they choose to seek help for insomnia in primary care, people have typically passed a certain threshold of symptomatology. Specifically, problems with fatigue and psychological distress are often the experiences that prompt people to seek help [9]. Some patients in the present study felt distressed because they thought they might get too little sleep. Obtaining a good night’s sleep is often a major concern for people with insomnia, and this concern helps maintain the disorder [55].

People’s confidence in their coping abilities affects their emotional reactions to difficult situations [53]. In the current study, some patients lacked confidence that the techniques would work for them or that they would be able to use what they learned; these patients expressed feelings of helplessness and failure. Patients with poor confidence in their coping ability might also feel less motivated to try to change their behaviors and to persist in behavioral change [54].

Sleep restriction is one of the most potent techniques in CBT-I [56]. In the present study, patients were mostly positive toward sleep restriction, but it could pose challenges. For instance, the limited sleep that came with the technique caused mental and physical tiredness, side effects that have been described previously [17, 57]. Like our study, an earlier study found that challenges to practicing sleep restriction also included experiences of negative impact on functioning, difficulties managing sleepiness prior to bedtime, boredom during extra hours awake, and changes in appetite/hunger [17]. In addition to these challenges, we found that sleep restriction could affect patients’ social life because of its inflexibility.

Patients used the techniques that felt right for them and adapted these techniques as needed. Like the findings of the current study, the findings of a qualitative meta-analysis of patients’ experiences of psychotherapy highlighted how important it was for patients to feel free to adjust techniques to their individual needs [18]. In our study, group leaders’ adaptation of sleep restriction to patients’ needs helped patients overcome barriers posed by this challenging technique. Patients who perceive fewer barriers to sleep restriction are more likely to continue practicing it [58].

Finally, the importance of adjusting techniques to the individual underscores the value of person-centered care. In person-centered care, one of the core competencies in nursing, the patient is viewed as an expert on her or his own experiences and everyday life [59]. Person-centered care can empower patients [60] by helping them feel able to make autonomous decisions about their self-management [61].

### Clinical implications

This study suggests ways in which the group treatment program for insomnia might be refined to better suit primary health care patients. Our findings indicate that certain aspects of the treatment program helped patients feel motivated and helped them achieve improvements. These included a group of people that shared the experience of insomnia, an involved and engaged leader, meeting at a convenient and trusted location, educational components, the sleep diary, regular meetings, and stepwise introduction and practice of techniques. Certain techniques seemed to be particularly helpful; for instance, sleep restriction, the relaxation exercise, power naps, reduction of hypnotic drugs, and techniques related to worry (worry time and identifying and arguing against negative thoughts).

Our findings also indicate that participation might be facilitated by fewer weekly treatment sessions. However, patients’ worries about future relapse suggest that follow-up sessions might be a helpful addition to the program.

Finally, the study provides insight into challenges related to treatment. Many of the challenges experienced by patients may be inevitable because they are related to insomnia itself (e.g., worry about sleep, tiredness, and a lack of confidence in the ability to sleep), and confronting them is a part of treatment. Other challenges are inherent in the techniques but can be minimized by adjusting the techniques to the individual. Still other challenges may arise from the group format. For instance, group leaders should keep in mind that comparing oneself to others is not always helpful. Whereas some people in the group may feel encouraged by such comparison, others can feel they have failed and even blame themselves for their perceived failure.

### Methodological discussion

A number of aspects of the study affected its trustworthiness. The atmosphere in the focus groups was relaxed, and it was clear that the patients felt familiar with each other. The participation of three to four patients in each focus group enabled each person to contribute to the discussion. However, larger focus groups might have offered more opportunities for active group interaction [62].

In qualitative analysis, data saturation is reached when new data no longer add novel information, but there is no consensus on how to determine when saturation has been achieved [63, 64]. In the current study, repeated interviews [65] were not carried out; we interviewed all the groups that were ongoing at the time of the study. However, the study had a relatively narrow aim, and sample specificity was high: all participants had experiences of the explored phenomenon. Moreover, we judge the dialogues to have been strong and data to have been rich within and across groups. Patients expressed a variety of experiences, characterized by both similarities and differences. We thus judge the study to have an adequate sample size in relation to information power [64]. Study findings were not presented to the study participants for validation [66].

In two of the five groups, the researcher who conducted the interview was also the group leader and had guided the participants through group treatment. This may be a limitation if patients felt they had to be particularly polite or focus on positive experiences. On the other hand, it may have helped patients feel secure because they knew the interviewer had followed their treatment process.

The prior understanding of two of the researchers was high, as they had previously led insomnia groups. Such prior understanding may have limited the analyses by affecting the interpretation of the data [67]. However, it may also have strengthened the study by conferring a deeper understanding of the explored context. To reduce the analytical limitations caused by prior understanding, all four authors participated actively in the analytical process, which involved ongoing discussion and reflection until consensus was reached. One of the authors of this study (KK) is bilingual; English is her native language. The others, like the patients, are native speakers of Swedish. KK and CS continuously discussed the coding with each other and collaborated to translate the condensed meaning units, quotations, codes, categories, subthemes, and themes. This increased the trustworthiness of the findings.

All interview participants were women, in part because of the distribution of insomnia in the general population [5] and in part because the majority of patients in the trial from which participants came were women (73%) [25]. It is possible that men would have experienced the group treatment differently or expressed their experiences in a different way.

The patients who were interviewed had participated voluntarily in and completed the group treatment. Many had waited for this treatment for more than 3 months, and none who had waited dropped out once they started. They were thus a selected and highly motivated group. Readers should take this into account when judging the transferability of the results. However, previous studies of treatments that encourage behavioral change have found experiences that are reflected in our findings. Thus, the findings of the current study may be relevant to other, similar treatment contexts.

## Conclusions

This study illuminated patients’ experiences of a nurse-led group treatment program for insomnia in primary health care. Patients described a process of motivation, change, and challenges. They experienced certain components as particularly important to motivation for behavioral change and for achieving improvements. Examples include meeting at a trusted and convenient location (the patients’ own primary health care center), being part of a group of people who shared similar experiences, gaining knowledge about sleep and insomnia, keeping a sleep diary, and practicing the sleep restriction technique. Moreover, this study highlights the importance of adapting treatment to differing needs and abilities and underscores the value of person-centered care. Finally, accessibility to treatment may be facilitated by fewer treatment sessions. These findings should be taken into account in future versions of the program.

## Abbreviations

CBT-I: Cognitive behavioral therapy for insomnia
G: Group
P: Patient
